# Revisiting the Flora of Saudi Arabia: Phytochemical and Biological Investigation of the Endangered Plant Species *Euphorbia saudiarabica*

**DOI:** 10.3390/metabo13040556

**Published:** 2023-04-13

**Authors:** Omer I. Fantoukh, Gadah A. Al-Hamoud, Fahd A. Nasr, Omer M. Almarfadi, Mohammed F. Hawwal, Zulfiqar Ali, Waleed A. Alobaid, Abdulaziz Binawad, Menwer Alrashidi, Fawaz Alasmari, Mohammad Z. Ahmed, Omar M. Noman

**Affiliations:** 1 Department of Pharmacognosy, College of Pharmacy, King Saud University, Riyadh 11451, Saudi Arabia; fnasr@ksu.edu.sa (F.A.N.); oalmarfadi@ksu.edu.sa (O.M.A.); mhawwal@ksu.edu.sa (M.F.H.); walobaid@ksu.edu.sa (W.A.A.); 438101803@student.ksu.edu.sa (A.B.); 438102630@student.ksu.edu.sa (M.A.);; 2 National Center for Natural Products Research, School of Pharmacy, The University of Mississippi, Oxford, MS 38677, USA; zulfiqar@olemiss.edu; 3 Department of Pharmacology and Toxicology, College of Pharmacy, King Saud University, Riyadh 11451, Saudi Arabia

**Keywords:** *Euphorbia saudiarabica*, phytochemicals, saudiarabicain F, cytotoxicity, apoptosis

## Abstract

*Euphorbia* plants have a significant place in traditional medicine due to their numerous therapeutic properties, including their anti-tumor effects, which have been observed in several species. In the current study, a phytochemical investigation of *Euphorbia saudiarabica* methanolic extract led to the isolation and characterization of four secondary metabolites from the chloroform (CHCl_3_) and ethyl acetate (EtOAc) fractions, which are reported for the first time in this species. One of the constituents, saudiarabicain F (**2**), is a rare C-19 oxidized ingol-type diterpenoid that has not been previously reported. The structures of these compounds were determined by extensive spectroscopic (HR-ESI-MS, 1D and 2D NMR) analyses. The anticancer properties of the *E. saudiarabica* crude extract, its fractions and its isolated compounds were examined against several cancer cells. The active fractions were evaluated for their effects on cell-cycle progression and apoptosis induction using flow cytometry. Furthermore, RT-PCR was employed to estimate the gene-expression levels of the apoptosis-related genes. It was demonstrated that the *E. saudiarabica* CHCl_3_ and EtOAc fractions suppressed the proliferation of the cancer cells. The MCF-7 cells were the most sensitive to both fractions, with IC_50_ values of 22.6 and 23.2 µg/mL, respectively. Notably, both fractions caused cell-cycle arrest in the G2/M phase of the treated MCF-7 cells. The inhibition of the MCF-7 cells’ proliferation was also linked with apoptosis induction by flow-cytometry analysis. Additionally, the activation of apoptosis by both fractions was demonstrated by an increase in the ratio of Bax to Bcl-2, with an increase in the expression of caspase-7. Among the isolated compounds, glutinol (**1**) showed potent activity against the MCF-7 cell line, with an IC_50_ value of 9.83 µg/mL. Our findings suggest that *E. saudiarabica* has apoptosis-inducing effects and shows promise as a potential source of new chemotherapeutic drugs.

## 1. Introduction

Cancer is one of the most significant health issues globally, with nearly 10 million deaths reported in 2020 [1]. The use of standard treatments, such as radiation, surgery and chemotherapy for cancer treatment is associated with significant side effects [2]. Therefore, there is an urgent need to develop novel anticancer compounds with fewer side effects, as well as to combat multi-drug resistance [3]. Since the dawn of human civilization, plants have been used as remedies to treat various diseases [4]. In recent years, medicinal plants, with their bioactive compounds, have played a significant role in discovering and developing therapeutic agents [5,6,7]. It was reported that approximately 50% of anticancer agents are derived from natural sources, either directly from nature or through semi-synthetic compounds [8,9].

Euphorbiaceae is a family of flowering plants with a rich history of ethnomedicinal use, in which *Euphorbia* is considered the third largest genus. Plants from this genus have traditionally been used to treat skin disease, venomous insect bites, abdominal pain, abdominal distention, trichiasis, and paralysis [10]. These therapeutic properties of *Euphorbia* species could be attributed to the presence of numerous secondary metabolites, such as triterpenes, diterpenes and flavonoids, which exhibit a wide range of biological activities. Regarding anticancer properties, several extracts or pure compounds from *Euphorbia* species have been reported to have potent cytotoxic activities against different cancer cells [11,12]. The species that have shown antiproliferative properties include *E. hirta* [13], *E. balsamifera* [14], *E. tirucalli* [15] and *E. fischeriana* [16]. Furthermore, anti-angiogenic, antitumor and genotoxic effects have also been observed in several *Euphorbia* species using in vitro and in vivo studies [17]. In their recently published review, Amtaghri et al. stated that around ten species of *Euphorbia* genus have been reported to have cytotoxic activity. Moreover, phytochemical studies of these species revealed that around twelve species contain several of the compounds responsible for their observed cytotoxic effects [18].

*Euphorbia saudiarabica* Fayed & Al-Zahrani is a succulent, spiny shrub or small tree, no more than 3 m tall, endemic to the southwestern corner of Saudi Arabia [19]. Only a few limited reports are available regarding the phytochemical composition and biological activities of this species. Recently, Muhsinah et al. isolated five novel 19-acetoxyingol compounds from the aerial parts of *E. saudiarabica.* They found that the isolated compounds exerted inhibitory effects on α-glucosidase and human P-glycoprotein and had weak cytotoxic activities [20]. To the best of our knowledge, this plant species has not been extensively explored for its chemical and pharmacological potential. In our continued quest to explore the flora of Saudi Arabia, the current study aimed to further investigate the phytochemical, antiproliferative and apoptotic properties of *E. saudiarabica*’s aerial parts.

## 2. Materials and Methods

### 2.1. Plant Material

The *E. saudiarabica* aerial parts were collected from the southern region of Saudi Arabia (18°09′510.5″, 41°61′951.6″) in April 2017 and were taxonomically identified by Dr. Ali Mohammed Alzahrani from the Biology Department, Al-Baha University, Saudi Arabia. A voucher specimen (no. AA123) was deposited in the herbarium of the Pharmacognosy Department.

### 2.2. Preparation of Extract

Air-dried powdered aerial parts of *E. saudiarabica* (400 g) were exhaustively extracted by maceration with MeOH (3L × 7) at room temperature. The resulting organic extracts were combined and filtered through Whatman paper no. 1. and then concentrated under reduced pressure at 45 °C to give a dry total extract of green residue weighing 70 g.

### 2.3. Isolation of Chemical Constituents

#### 2.3.1. Liquid–Liquid Partition

A 70 g of *E. saudiarabica* methanol (MeOH) extract was suspended in 500 mL of water and successively fractionated by chloroform (CHCl_3_), ethyl acetate (EtOAc) and *n*-butanol (*n*-BuOH), respectively, using 500 mL. This was repeated three times for each organic solvent. A rotary evaporator (45 rpm and 45 °C) was used to concentrate the filtrates. The yields of the dried fractions were 1.13 g (CHCl_3_ fraction), 2.20 g (EtOAc fraction) 9.20 g, (*n*-BuOH fraction) and 57 g (aqueous fraction). The dried portions were then collected in vials and kept at –20 °C until needed.

#### 2.3.2. Isolation of Compounds from CHCl_3_ Fraction

The CHCl_3_ fraction (1.13 g) was fractionated using flash chromatography (Isolera 4). It was first eluted with *n*-hexane (100%), followed by a mixture of *n*-hexane/EtOAc with an increasing polarity up to 100% EtOAc. The eluent was collected in 15-mL test tubes. The fractions were pooled into 13 fractions based on TLC similarities. Subfraction 11 showed a clear spot (RF = 0.32) using NP-TLC developed in *n*-hexane/EtOAc (80:20) and sprayed by anisaldehyde–sulfuric acid and afforded compound **1** (12 mg).

#### 2.3.3. Isolation of Compounds from EtOAc Fraction

The EtOAc fraction (2.20 g) was fractionated by flash chromatography (Isolera 4). It was first eluted with *n*-hexane (100%), followed by a mixture of *n*-hexane/MeOH with an increasing polarity up to 80% MeOH. The eluent was collected in 15-mL test tubes, which were pooled into 8 subfractions based on TLC similarities. Subfraction 8 was subjected to purification using RP-column eluted with H_2_O (100%), followed by a mixture of H_2_O/MeOH with an increasing MeOH ratio up to 80% MeOH. The eluent was collected in test tubes (2 mL each), which were pooled into 10 subfractions based on the TLC similarity. Sub-fractions 5, 7, and 10 showed clear spots (RF = 0.50, 0.29, 0.26, respectively) using RP-TLC developed by H_2_O/MeOH (50:50) and were afforded compounds **2** (8.3 mg), **3** (54.3 mg) and **4** (8.5 mg), respectively.

### 2.4. Spectral Analysis of Isolated Compounds

The NMR spectra were run on a Bruker Avance 500-MHz spectrometer (Bruker, Germany) operating at 500 MHz (^1^H-NMR) and 125 MHz (^13^C-NMR). The samples were dissolved in spectroscopic-grade CDCl_3_ (compound **1**) and DMSO-d6 (compounds **2**–**4**).

Glutinol (**1**): white powder; MS/ESI: *m/z* 426, calculated for C_30_H_50_O, 449 [M+Na]^+^; ^1^H NMR data (500 MHz, CDCl_3_) δH (ppm): 3.52 (1H, br s, H-3), 5.66 (1H, m, H-6), 1.07 (3H, s, H-23), 1.17 (3H, s, H-24), 0.87 (3H, s, H-25), 1.03 (3H, s, H-26), 1.12 (3H, s, H-27), 1.19 (3H, s, H-28), 1.02 (3H, s, H-29), 0.98 (3H, s, H-30); ^13^C NMR (125 MHz, CDCl_3_) δc (ppm): 18.22 (C-1), 27.82 (C-2), 76.36 (C-3), 40.84 (C-4), 141.61 (C-5), 122.09 (C-6), 23.64 (C-7), 47.44 (C-8), 34.85 (C-9), 49.69 (C-10), 34.61 (C-11), 30.36 (C-12), 39.30 (C-13), 37.84 (C-14), 32.04 (C-15), 36.02 (C-16), 30.10 (C-17), 43.06 (C-18), 35.08 (C-19), 28.26 (C-20), 32.40 (C-21), 38.96 (C-22), 25.47 (C-23), 28.95 (C-24), 16.22 (C-25), 19.63 (C-26), 18.44 (C-27), 31.94 (C-28), 33.11 (C-29), 32.08 (C-30). The NMR data were comparable to those reported in the literature and named as glutinol [21].

Saudiarabicain F (**2**): amorphous white powder; MS/ESI: *m/z* 382.1979, calculated for C_20_H_30_O_7_, 405.1890 [M+Na]^+^; ^1^H NMR data (500 MHz, DMSO-d6) δH (ppm): 2.54 (1H, dd, H-1a), 1.47 (1H, d, H-1b), 2.12 (1H, m, H-2), 4.21 (1H, d, H-3), 5.60 (1H, s, H-5), 3.93 (1H, d, H-7), 3.32 (1H, dd, H-8), 1.18 (1H, dd, H-9), 0.69 (1H, dd, H-11), 3.34 (1H, dd, H-12), 2.67 (1H, dq, H-13), 0.89 (3H, d, H-16), 1.83 (3H, d, H-17), 1.07 (3H, s, H-18), 3.58 (1H, d, H-19a), 3.51 (1H, d, H-19b), 1.01 (3H, d, H-20); ^13^C NMR (125 MHz, DMSO-d6) δc (ppm): 31.33 (C-1), 30.87 (C-2), 74.85 (C-3), 76.11 (C-4), 116.63 (C-5), 141.64 (C-6), 77.69 (C-7), 69.21 (C-8), 27.84 (C-9), 23.20 (C-10), 33.66 (C-11), 68.34 (C-12), 42.69 (C-13), 210.73 (C-14), 71.51 (C-15), 17.19 (C-16), 17.87 (C-17), 25.57 (C-18), 63.01 (C-19), 13.93 (C-20). The NMR data were similar to saudiarabicain E reported in the literature, with hyrdroxy rather than benzyl and acetyl groups [20]; the compound was named saudiarabicain F. The relevant MS/ESI and 1D and 2D NMR data of saudiarabicain F are illustrated in the Supplementary Material (Figures S1–S6).

Quercetin-3-*O*-α-L-rhamnopyranoside (**3**): amorphous yellow powder; MS/ESI: *m/z* 448 calculated for C_21_H_20_O_11_, 471 [M+Na]^+^; ^1^H NMR data (500 MHz, DMSO-d6) δH (ppm): 6.17 (1H, d, H-6), 6.34 (1H, d, H-8), 7.30 (1H, d, H-2′), 6.88 (1H, d, H-5′), 7.28 (1H, dd, H-6′), 5.32 (1H, d, H-1″), 4.19 (1H, dd, H-2″), 3.72 (1H, dd, H-3″), 3.31 (1H, dd, H-4″), 3.39 (1H, dd, H-5″), 0.91 (3H, d, H-6″); ^13^C NMR (125 MHz, DMSO-d6) δc (ppm): 157.22 (C-2), 134.89 (C-3), 178.31 (C-4), 161.89 (C-5), 98.55 (C-6), 164.80 (C-7), 93.42 (C-8), 157.96 (C-9), 104.49 (C-10), 121.63 (C-1′), 115.03 (C-2′), 145.11 (C-3′), 148.50 (C-4′), 115.58 (C-5′), 121.52 (C-6′), 102.22 (C-1″), 70.77 (C-2″), 70.70 (C-3″), 71.91 (C-4″), 70.57 (C-5″), 16.32 (C-6″). The NMR data were comparable to those reported in the literature and identified as quercetin-3-*O*-α-L-rhamnopyranoside [22].

Kaempferol-3-*O*-α-L-rhamnopyranoside (**4**): amorphous yellow powder; MS/ESI: *m/z* 432 calculated for C_21_H_20_O_10_, 455 [M+Na]^+^; ^1^H NMR data (500 MHz, DMSO-d6) δH (ppm): 6.18 (1H, d, H-6), 6.35 (1H, d, H-8), 7.74 (2H, d, H-2′, H-6′), 6.91 (2H, d, H-3′, H-5′), 5.35 (1H, d, H-1″), 4.19 (1H, dd, H-2″), 3.68 (1H, dd, H-3″), 3.48 (1H, m, H-4″), 3.34 (1H, m, H-5″), 0.89 (3H, d, H-6″); ^13^C NMR (125 MHz, DMSO-d6) δc (ppm): 157.95 (C-2), 134.86 (C-3), 178.28 (C-4), 161.91 (C-5), 98.57 (C-6), 164.80 (C-7), 93.46 (C-8), 157.26 (C-9), 104.52 (C-10), 121.30 (C-1′), 130.57 (C-2′, 6′), 115.20 (C-3′, 5′), 160.29 (C-4′), 102.19 (C-1″), 70.58 (C-2″), 70.77 (C-3″), 71.83 (C-4″), 70.71 (C-5″), 16.31 (C-6″). The NMR data were comparable to those reported in the literature and identified as kaempferol-3-*O*-α-L-rhamnopyranoside [23]. 

### 2.5. Cell Culture and MTT Cytotoxicity Assay

Non-cancerous cell line (HUVEC) was obtained from ATCC (16549; cat# PCS-100-010, Manassas, VA, USA), while the cancer-cell lines A549, MCF-7, and LoVo were obtained from DSMZ Leibniz Institute (German Collection of Microorganisms and Cell Cultures Braunschweig, Germany).

Cell viability was determined by the 3-(4,5-dimethylthiazol-2-yl)-2,5-diphenyl tetrazolium bromide (MTT) assay, as previously described [10]. Different cancer cells, including lung (A549), colon (LoVo) and breast (MCF-7) cancer cells, as well as normal HUVEC cells, were employed in this study. In brief, 100 µL of cells (5 × 10^4^ cells/mL) was seeded in 96-well plates and incubated at 37 ^∘^C in a 5% carbon-dioxide incubator. After 24 h of incubation, the cells were treated with various concentrations of plant crude extracts and their fractions: CHCl_3_, EtOAc, BuOH and aqueous. After 48 h, 10 µL of MTT solution (5 mg/mL) (Invitrogen, USA) was added to each well and the plates were further incubated for 2 to 4 h. Formazan product was dissolved in 100 μL of acidified isopropanol and the absorbance was measured at 570 nm. Percentages of cell viability and IC_50_ values were calculated according to the equation:

% Cell viability = Absorbance of treated sample/Absorbance of control × 100

### 2.6. Cell-Cycle Analysis

The DNA-content analysis was performed as previously described [24]. Briefly, MCF-7 cells were treated with CHCl_3_ and EtOAc fractions at 10 and 20 μg/mL for 24 h. Before harvesting, cells were harvested, washed with phosphate-buffered saline (PBS) and fixed in 70% ethanol at 4 °C. Next, cell pellets were washed once with PBS, resuspended in propidium iodide and RNase and then incubated for half an hour in the dark. Cell-cycle analysis was performed with flow cytometry (Cytomics FC 500; Beckman Coulter, Brea, CA, USA). Data collection and analysis were performed using CXP software 3.0.

### 2.7. Annexin V-FITC Staining Assay

The apoptotic effect of CHCl_3_ and EtOAc fractions was determined by an Annexin V-FITC/PI assay and measured on a flow cytometer (Cytomics FC 500; Beckman Coulter, Brea, CA, USA). In brief, MCF-7 cells were treated with different concentrations of each fraction (10 and 20 µg/mL) in 6-well plates for 24 h. Both untreated and treated cells were harvested and washed with PBS. The cell pellets were then resuspended in 100 µL of Annexin binding buffer. Subsequently, 5 μL of propidium iodide and Annexin V-FITC were added to the resuspended cells and incubated for 20–30 min at room temperature in the dark. The samples were then diluted with annexin buffer (400 µL) prior to being subjected to flow-cytometry analysis.

### 2.8. RNA Extraction and RT-PCR

Total RNA was extracted using the TRIzol (Invitrogen, Waltham, MA, USA) procedure from treated and untreated cell samples and the amount of RNA was determined with a NanoDrop ND-1000 spectrophotometer (Thermo Fisher, Waltham, MA, USA). SuperScript Vilo cDNA synthesis (Invitrogen, USA) was used to create cDNA as follows: 1 μg of total RNA, 4 μL of the 5× Vilo reaction mix, 2 μL of the 10× SuperScript mix and RNA-free water were added to make a total volume of 20 μL. We used β-actin as an internal control and a semiquantitative PCR was used to measure the expression of Bax, Bcl-2 and caspase-7 in MCF-7 cells. The final volume (20 μL) of the RT-PCR mixture contained 4 μL of 5X FIRE pol Master Mix, 10 pmol of each specified primer and double-distilled water. The sequences of the primers used were as follows: Bax, F: 5′-CGGGTTGTCGCCCTTTTCTA-3′, R: 5′-AAAGTAGGAGAGGAGGCCGT-3′; Bcl-2, F: 5′-TGATGCCTTCTGTGAAGCAC-3′, R: 5′-ACAGGCGGAGCTTCTTGTAA-3′; caspase-7, F: 5′-AGTGACAGGTATGGGCGTTC-3′, R: 5′-TCCATGGCTTAAGAGGATGC-3′; β-actin; F: 5′-ACTGGGACGACATGGAGAAAA-3′, R: 5′-GAGGCGTACAGGGATAGCAC-3′. The 20-µL final amplification products were run on 1.2% agarose gel stained with ethidium bromide and a gel image was captured with LICOR gel doc.

### 2.9. Statistical Analysis

The data are displayed as mean ± standard deviation (SD). The differences between the means of the various groups were examined using Student’s *t*-test (OriginPro software, USA). A *p*-value < 0.05 was considered statistically significant.

## 3. Results

### 3.1. Isolation of Secondary Metabolites from the MeOH Extracts from Aerial Parts of E. saudiarabica

**Figure 1:**
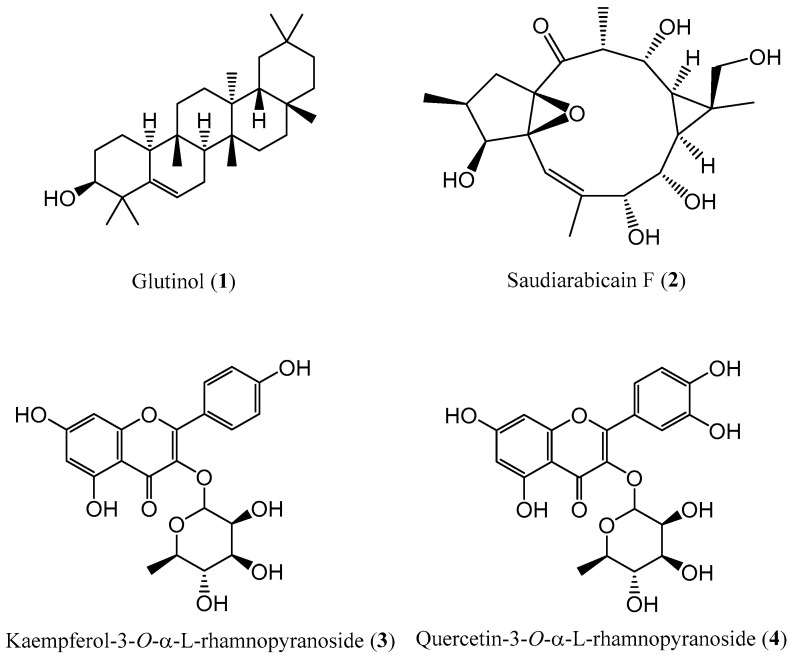
Chemical structures of phytochemicals isolated from *Euphorbia saudiarabica* aerial parts.

The phytochemical investigation of the MeOH extracts from the aerial parts of the *E. saudiarabica* resulted in the isolation and characterization of one compound from the CHCl_3_ fraction and three secondary metabolites from the EtOAc fraction (Figure 1). Their chemical structures were established using NMR and MS, as well as a comparison with data obtained from the available literature. Specifically, the obtained compounds were a triterpenoid derivative, a macrocyclic ingol-type diterpenoid and two flavonoids, which are here reported in this plant species for the first time and in agreement with the chemotaxonomic profile of the genus *Euphorbia*. Among these constituents, saudiarabicain F (**2**) represents a rare class of C-19 oxidized ingol-type diterpenoid, which was undescribed previously.

**Figure 2:**
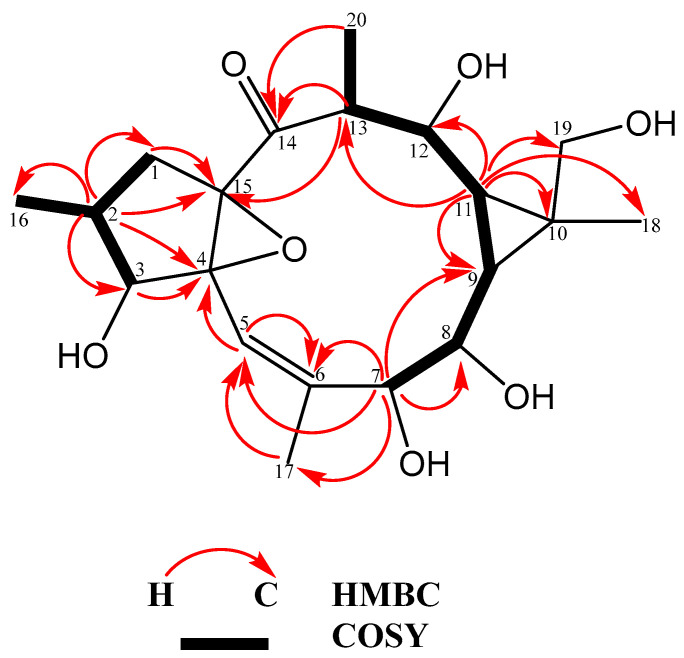
COSY and HMBC correlations of saudiarabicain F (**2**) isolated from *E. saudiarabica*.

The saudiarabicain F (**2**) was obtained as a white powder with the molecular formula C_20_H_30_O_7_, as determined by HR-ESI-MS (*m/z* 382.1992), corresponding to five degrees of unsaturation. The NMR data of compound **2**, when compared to the previously reported ingol-type diterpenoid, saudiarabicain E, revealed that the only difference between these two compounds was the presence of hydroxy rather than benzyl and acetyl groups in C-3, C-7, C-8, C-12 and C-19 [20]. Specifically, the ^13^C and ^1^H NMR spectra revealed the presence of a singlet keto group (δ_C_ 210.73) and a trisubstituted double bond (δ_C_ 141.64, 116.63; δ_H_ 5.60). These observations reflect two out of five indices of degrees of unsaturation, which supported a tricyclic carbon skeleton for compound **2**. In addition to these signals, the ^13^C and DEPT-135 NMR spectra displayed signals for the remaining skeletal carbons, including four methyls (one olefinic), two methylenes (one oxygenated), eight methines (four oxygenated) and three non-protonated carbons (two oxygenated). The structure of compound **2** was further established by an extensive analysis of the HSQC, HMBC and ^1^H-^1^H COSY spectra (Figure 2). The ^1^H-^1^H COSY correlations of H_2_-1/H-2/H-3 and H-2/H_3_-16, together with H-7/H-8/H-9/H-11/H-12/H-13/H_3_-20, suggested the two spin systems of compound **2**, while the HMBC correlations from H-13 to C-15 and H-2 to C-15 established the connections between these two fragments. Furthermore, the HMBC correlations from H-2 to C-4 and C-15 suggested the 4,15-expoxy ring. Additionally, the HMBC correlations from H_3_-18 to C-9, C-10 and C-19, as well as those from Ha-19 and Hb-19 to C-10 and C-11, revealed a cyclopropane ring, located at C-9 and C-11. In addition, the HMBC correlations from H-13 and H_3_-20 to δ_C_ 210.73 suggested that the carbonyl was linked to C-13. The relative configuration of compound **2** was assigned through the analysis of its ^1^H NMR coupling constants. Specifically, the coupling constant of *J*_2,3_ = 8.3 Hz and the chemical shifts of H_2_-1 (δ_H_ 2.54 and 1.47) indicated that the H-2 and H-3 protons were in an α-orientation [25]. Up to this point, the configuration of the H-7, H-8 and H-12 in all the ingol diterpenoids isolated from this plant were in the β-orientation. In addition, the coupling constants for H-7 (*J* = 1.0 Hz), H-8 (*J* = 9.5, 1.0 Hz), H-12 (*J* = 10.5, 3.7 Hz) and H-13 (*J* = 7.3, 3.7 Hz) were similar to those reported for saudiarabicain E and labeled arbitrarily as β orientation [20]. Moreover, the coupling constants for H-9 (*J* = 9.5, 9.5) and H-11 (*J* = 10.5, 9.5) were similar to those reported for saudiarabicain E and labeled arbitrarily as α orientation. As a result, the absolute configuration of compound **2** was assigned as (2S, 3S, 4S, 7R, 8S, 9S, 10R, 11R, 12R, 13R, 15R).

### 3.2. Antiproliferative and Apoptotic Activity of E. saudiarabica

**Figure 3:**
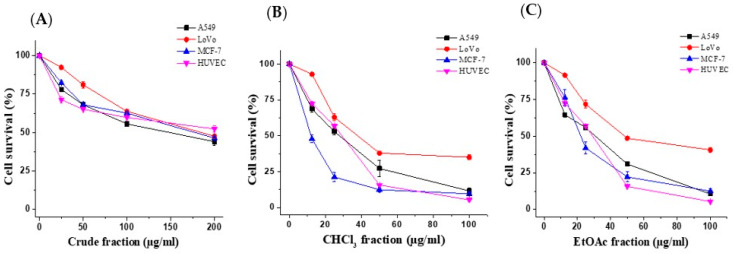
Cell viability of different cells following treatment with *E. saudiarabica* crude extract, CHCl_3_ and EtOAc fractions. The cell viability was measured by MTT assay. Cells were treated with different concentrations of crude extract (**A**), CHCl_3_ (**B**) or EtOAc (**C**) fractions. Data are presented as the mean ± standard deviation (SD) from three independent experiments.

The effects of the *E. saudiarabica* crude extract and its four fractions on the proliferation of the A549, LoVo, MCF-7 and HUVEC cells were examined using the MTT cell-viability assay. Figure 3A shows that the crude extract dose-dependently inhibited the proliferation of the tested cells. The crude extract was fractionated into CHCl_3_, EtOAc, *n*-BuOH and water-soluble fractions. Of the four, the CHCl_3_ and EtOAc fractions caused the most significant proliferation inhibition (Figure 3B,C). As presented in Table 1, the lowest IC_50_ was obtained with the CHCl_3_ and EtOAc fractions for all the tested cells. Remarkably, we found that the MCF-7 cell line was the most sensitive to the CHCl_3_ and EtOAc fractions with IC_50_ of 22.6 and 23.2 µg/mL, respectively. Our results also revealed that the IC_50_ values of the CHCl_3_ and EtOAc fractions were slightly higher (28.8 and 29 µg/mL) against the normal HUVEC cells. Hence, the CHCl_3_ and EtOAc fractions were selected for further investigation against MCF-7 cells.

**Figure 4:**
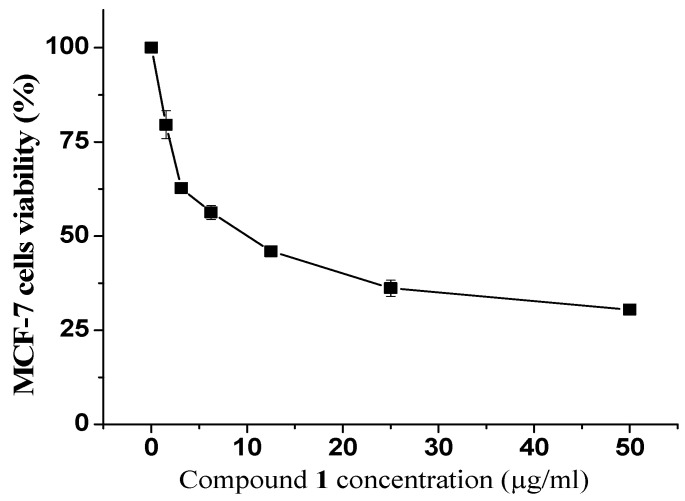
Viability percentages of MCF-7 cells treated with compound **1.** Cells were treated with the indicated concentration of compound **1** for 48 h and their viability was assessed by MTT assay. Results are expressed as Avg ± SD of three experiments.

Additionally, the cytotoxic effects of the isolated compounds on the MCF-7 cells were evaluated. Among all the isolated compounds, we found that compound **1** exhibited a strong antiproliferative activity. As shown in (Figure 4), the MCF-7 cells’ survival decreased along with increases in the concentration of compound **1**. The reported IC_50_ value for compound **1** was 9.8 µg/mL, while the remaining compounds were considered inactive at the highest tested concentration (50 µg/mL) (Table 2).

### 3.3. Cell-Cycle Analysis

**Figure 5:**
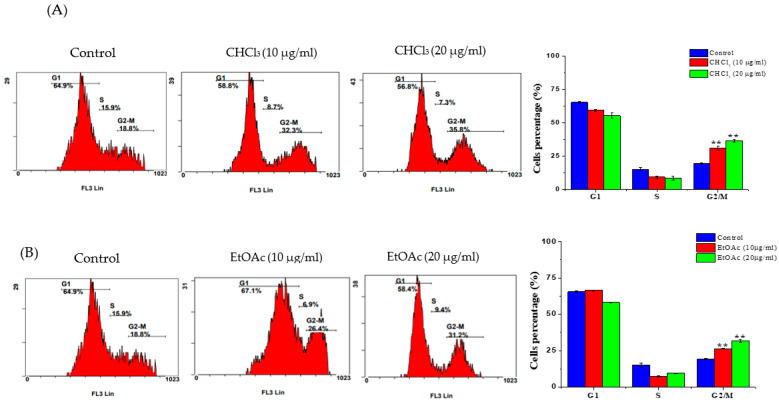
Cell-cycle progression of MCF-7 cells after exposure to (**A**) CHCl_3_ and (**B**) EtOAc fractions of *E. saudiarabica*. The MCF-7 cells were treated with 10- and 20-μg/mL concentrations for 24 h and the distribution of cells in various stages was evaluated by measuring DNA content. Results showed that both fractions induced G2/M cell-cycle arrest in a dose-dependent manner. The experiments were performed in triplicate. Data are reported as Avg ± SD. ** *p* ≤ 0.01 vs. control.

Propidium-iodide staining was employed to study the distribution of the cell-cycle phases of the fraction-treated cells. As displayed in Figure 5A, upon the treatment of the MCF-7 cells with 10 and 20 µg/mL of the CHCl_3_ fraction, there was a significant increase in the percentage of G2/M cells (which increased to 31.15 ± 1.62 and 36.6 ± 1.13, respectively) compared to the untreated cells (19.35 ± 0.5). Similarly, the treatment of the MCF-7 cells with 10 and 20 µg/mL of EtOAc caused a significant increase in the percentage of cells in the G2/M phase (with increases to 26.3 ± 0.14 and 31.8 ± 0.84, respectively) compared to the untreated cells (Figure 5B).

### 3.4. Apoptosis Detection

**Figure 6:**
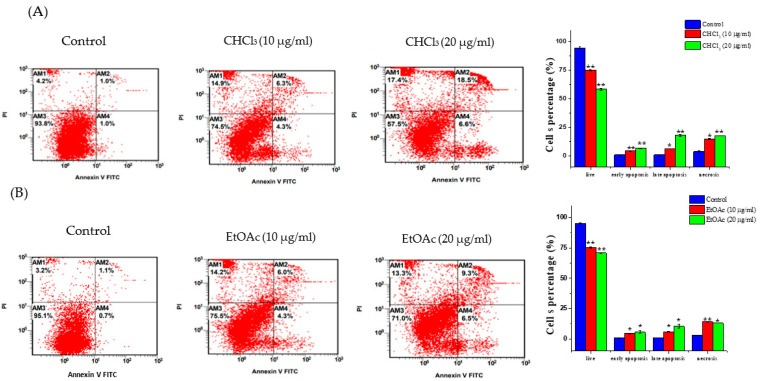
Effects of *E. saudiarabica* CHCl_3_ and EtOAc fractions on stages of apoptosis. (**A**) The MCF-7 cells were incubated with 10 and 20 μg/mL of CHCl_3_ fraction for 24 h and were then collected and stained with Annexin V/PI, followed by FACS analysis. The bars display FACS-quantification analysis. (**B**) The FACS and bar graphs showing the percentages of MCF-7 cells treated with 10 and 20 μg/mL of CHCl_3_ fraction for 24 h. Data are mean ± SEM of three independent experiments, where * denotes *p* ≤ 0.05 and ** denotes *p* ≤ 0.01 vs. control, as measured by Student’s *t*-test.

Apoptosis and necrosis are the two mechanisms that are most heavily involved in cell death. Therefore, the effects of the CHCl_3_ and EtOAc fractions (10 and 20 µg/mL) on apoptosis/necrosis induction in the MCF-7 cells were evaluated. The treatment of the MCF-7 cells with 10 µg/mL of the CHCl_3_ and EtOAc fractions increased the number of cells undergoing early (4.25 ± 0.07% and 4.55 ± 0.33%, respectively) and late apoptosis (6.2 ± 0.14% and 5.9 ± 0.14%, respectively) compared to the untreated cells (0.95 ± 0.14% and 1.05 ± 0.07%, respectively). An increase in the percentage of necrotic cells was also observed following the treatments with the CHCl_3_ and EtOAc fractions (14.65 ± 0.35% and 17.35 ± 0.7%, respectively) compared to the control cells (3.8 ± 0.56%) (Figure 6A,B). Additionally, increasing the concentrations of the CHCl_3_ and EtOAc fractions to 20 µg/mL resulted in significant increases in the number of cells undergoing apoptosis and necrosis.

### 3.5. RT-PCR

**Figure 7:**
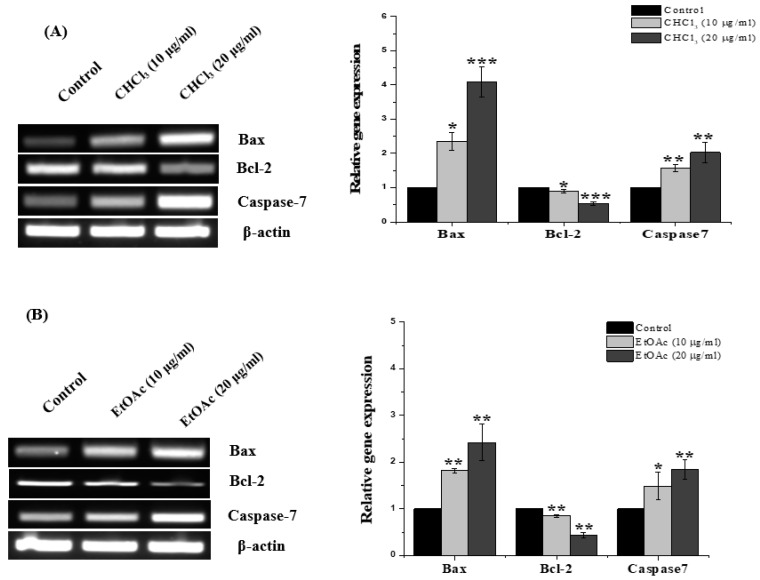
Gene-expression analysis of Bcl-2, Bax and caspase-7 after incubation of MCF-7 cells with 10 and 20 μg/mL of (**A**) *E. saudiarabica* CHCl_3_ fraction and (**B**) *E. saudiarabica* EtOAc fraction. Histograms showing densitometry analysis, in which band intensity was measured and normalized to that of β-actin. Data shown are means ± SD (*n* = 3) (* *p* < 0.05, ** *p* < 0.01 and *** *p* < 0.001 vs. control).

To determine the apoptotic actions of the CHCl_3_ and EtOAc fractions against the MCF-7, the gene-expression levels of the proapoptotic (Bax and caspase-7) and antiapoptotic (Bcl-2) genes were estimated after exposing the cells to 10 and 20 μg/mL of the fractions. The mRNA expression of the antiapoptotic Bcl-2 was reduced after exposure to the CHCl_3_ and EtOAc fractions (Figure 7A,B). In contrast, the proapoptotic Bax significantly increased (2.34 ± 0.46 and 4.09 ± 0.44-fold), as did he caspase-7 (1.57 ± 0.11 and 2.02 ± 0.2-fold), following treatment with the CHCl_3_ fraction at 10 and 20 μg/mL, respectively. In the same manner, the upregulation of Bax (1.81 ± 0.04 and 2.41 ± 0.39-fold) and caspase-7 (1.48 ± 0.29 and 1.83 ± 0.21-fold) was observed when using the EtOAc treatment at the indicated concentrations.

## 4. Discussion

Several studies have shown the beneficial effects of natural phytochemicals on the treatment of various illnesses, including cancer [26]. In this study, as part of our continuing search for the cytotoxic properties of *Euphorbia* species grown in Saudi Arabia [10], we report, for the first time, the anticancer activity of *E. saudiarabica* against a panel of human cancer cells. The mechanism through which *E. saudiarabica* inhibits cancer-cell proliferation was also explored in this study. According to the obtained IC_50_ values (Table 1), the CHCl_3_ and EtOAc fractions met the criteria of the American National Cancer Institute, according to which IC_50_ values of less than 30 μg/mL indicate a promising extract [27]. The MCF-7 cells were the most sensitive to the CHCl_3_ and EtOAc fractions. Therefore, they were selected for the assessment of the inhibition mechanisms of both fractions. The IC_50_ value obtained in this study was very close to that reported by Kwan et al. [13], who found that the MeOH extract from *E. hirta* inhibited MCF-7-cell proliferation with an IC_50_ value of 25.26 g/mL. Our findings are also in line with the previously mentioned results obtained by El-Hawary et al. [28], who studied the cytotoxic potential of several *Euphorbia* species, which demonstrated strong activity against MCF-7 cells. Another *Euphorbia*-species study [29] found that a *n*-BuOH extract from *E. tirucalli* inhibited MCF-7-cell proliferation, with an IC_50_ value of 15 μg/mL, which is close to the value we obtained in this study using the same cells. In terms of IC_50_ values, the CHCl_3_ and EtOAc fractions had lower cytotoxic effects against normal HUVEC cells (IC_50_ = 28.8 and 29 μg/mL) than the MCF-7 cells (22.6 and 23.2 μg/mL). However, the extracts tested in the current research had some effects on cancer cells and normal HUVEC cells. This was in line with numerous studies that have documented the toxicity of chemically therapeutic drugs and plant extracts [30,31,32].

The National Cancer Institute (NCI) criteria recommends an IC_50_ value lower than 4 μg/mL to categorize pure compounds as promising cytotoxic compounds [33]. Herein, compound **1** demonstrated an IC_50_ value that was close to the borderline of this criterion, suggesting a possible role for this compound in the cytotoxicity of *E. saudiarabica*. In fact, the possible role of natural triterpenoids and their derivatives in the treatment of breast cancer has been reported. These antineoplastic properties of triterpenoids in breast cancer could be attributed to a plethora of biological activities, including cell-cycle regulation, cell-proliferation inhibition and apoptosis induction [34].

Many chemotherapeutic drugs block the proliferative phase of the cell cycle. Therefore, regulating cell-cycle progression is considered a promising cancer-treatment strategy [35]. Moreover, several studies emphasized the importance of phytochemicals and their impact on cell-cycle regulation [36]. Here, the two fractions caused cell death through the induction of cell-cycle arrest in the G2/M phase, suggesting that both fractions prevent cells from entering the mitosis phase. This characteristic is linked with the mechanism of the well-known anticancer drug, paclitaxel [37]. The cell-cycle-pattern arrest observed in the G2/M phase in this study is similar to those in several other studies of the same *Euphorbia* species, including *E. hirta* [13], *E. erythradenia* [38], *E. lunulata* [39] and *E. cactus* [10], with different cancer cells. Standard cancer treatments are intended to initiate apoptotic cell death, which is a fundamental process in preventing cancer-cell growth [40,41,42]}. The quantitative analysis using flow cytometry in the present work demonstrated that both the CHCl_3_ and the EtOAc fractions induced apoptotic cell death and a noticeable increase in necrotic cells. This suggests that apoptosis was one of the cell-death modes caused by both fractions. This finding is consistent with other reports demonstrating that the anticancer effects of several *Euphorbia* species on different cancer-cell lines were due to their apoptosis-inducing activity [10,13,38,43]. To confirm the observed apoptotic effects of both fractions, RT-PCR was used to evaluate the levels of the Bcl-2-family genes that are involved in apoptosis. It is well-known that Bcl-2-family genes play a crucial role in apoptosis regulation as either suppressors, as in the case of Bcl-2, or promoters, as in the case of Bax [44]. It was observed that the *E. saudiarabica* CHCl_3_ and EtOAc fractions were involved in the overexpression of Bax and the downregulation of Bcl-2. In addition, Bax expression, which leads to apoptosis induction, has also been correlated with caspase activation, which ultimately leads to programmed cell death [45,46]. Here, we found that executioner caspase-7 was upregulated, along with Bax, in the induction of apoptosis by the *E. saudiarabica* extracts.

## 5. Conclusions

This study provided insights into the chemical constituents of *E. saudiarabica* species that have not been widely investigated in terms of their chemical components and anticancer activities. In this study, four secondary metabolites (**1**–**4**) were isolated and identified from *E. saudiarabica* for the first time. In particular, saudiarabicain F (**2**), which was not described previously. We demonstrated that the *E. saudiarabica* extracts exhibited potent antiproliferative activities and inhibited the proliferation of different cancer cells. Furthermore, our investigation revealed that the *E. saudiarabica* CHCl_3_ and EtOAc fractions caused cell-cycle arrest in the G2/M phase in the MCF-7 cells. We also provided evidence that both fractions induce apoptosis through the upregulation of Bax and the downregulation of Bcl-2. Of the isolated compounds, only glutinol (**1**) exhibited remarkable cytotoxicity against the MCF-7 cells. To obtain a comprehensive understanding of *E. saudiarabica*’s anticancer properties, further investigation is required to examine the underlying mechanism in other cancer-cell lines, as well as in animal models.

## Figures and Tables

**Table 1:** IC_50_ values in μg/mL for *E. saudiarabica* fractions obtained by MTT assay with different cancer-cell lines, as well as normal HUVEC cells treated for 48 h.

	A549	LoVo	MCF-7	HUVEC
MeOH extract	150.6 ± 4.6	184.3 ± 5.1	178.1 ± 3.4	>200
CHCl_3_ fraction	27.3 ± 1.7	37.8 ± 0.7	22.6 ± 0.6	28.8 ± 0.3
EtOAc fraction	30.6 ± 1.1	48.0 ± 1.1	23.2 ± 0.8	29.0 ± 0.3
BuOH fraction	NA	NA	NA	NA
H_2_O fraction	NA	NA	NA	NA
Doxorubicin	1.79 ± 0.03	0.95 ± 0.04	1.3 ± 0.1	1.2 ± 0.4

**Table 2:** Cytotoxic activities of compounds isolated from *E. saudiarabica*.

Compounds	MCF-7 Cells IC_50_ (µg/mL)
Glutinol (**1**)	9.83 ± 0.55
Saudiarabicain F (**2)**	>50
Quercetin-3-*O*-α-L-rhamnopyranoside (**3**)	>50
Kaempferol-3-*O*-α-L-rhamnopyranoside (**4**)	>50
Doxorubicin	1.3 ± 0.1

## Data Availability

All data generated or analyzed in the current study are included in this article.

## Supplementary Materials

The following supporting information can be downloaded at: https://www.mdpi.com/article/10.3390/metabo13040556/s1. Figure S1: The ^1^H NMR spectrum of compound **2** in DMSO-d6; Figure S2: The ^13^C NMR spectrum of compound **2** in DMSO-d6; Figure S3: The DEPT-135 NMR spectrum of compound **2** in DMSO-d6; Figure S4: The HSQC spectrum of compound **2** in DMSO-d6; Figure S5: The COSY spectrum of compound **2** in DMSO-d6; Figure S6: The HMBC spectrum of compound **2** in DMSO-d6; Figure S7: HR-ESI-MS spectrum of compound **2** in the positive mode; Figure S8: HR-ESI-MS spectrum of compound **2** in the negative mode.

## Abbreviations

MeOH	Methanol
EtOAc	Ethyl acetate
*n*-BuOH	*n*-butanol
DMSO	Dimethyl sulfoxide.
MTT	3-[4,5-dimethylthiazole-2-yl]-2,5-diphenyltetrazolium bromide.
MCF7	Michigan Cancer Foundation-7.
V-FITC	V-fluorescein Isothiocyanate.
RT-PCR	Reverse transcription polymerase chain reaction
