# Supplementary material for: Revisiting the Flora of Saudi Arabia: Phytochemical and Biological Investigation of the Endangered Plant Species Euphorbia saudiarabica
Source: Metabolites. 2023 Apr 13;13(4):556. doi: 10.3390/metabo13040556 (PMC10144502; doi:10.3390/metabo13040556)
Supplement: Supplementary file 1 [file metabolites-13-00556-s001.zip › metabolites-2306158-supplementary.pdf]

Supplemnetary Materials:

Revisiting the Flora of Saudi Arabia: Phytochemical and Biological Investigation of an Endangered Plant Species *Euphorbia saudiarabica*

Omer I. Fantoukh [1,*,†], Gadah A. Al-Hamoud [1,*,†], Fahd A. Nasr [1], Omer M. Almarfadi [1], Mohammed F. Hawwal [1], Zulfiqar Ali [2], Waleed A. Alobaid [1], Abdulaziz Binawad [1], Menwer Alrashidi [1], Fawaz Alasmari [3], Mohammad Z. Ahmed [1] and Omar M. Noman [1]

[1] Department of Pharmacognosy, College of Pharmacy, King Saud University, P.O. Box 2457, Riyadh 11451, Saudi Arabia

[2] National Center for Natural Products Research, School of Pharmacy, The University of Mississippi, Oxford, MS 38677, USA

[3] Department of Pharmacology and Toxicology, College of Pharmacy, King Saud University, Riyadh 11451, Saudi Arabia

*** Authors to whom correspondence should be addressed.**

[†] These authors contributed equally to this work.

Table of Contents:

Figure S1: The ^{1}H NMR spectrum of compound **2** in DMSO-d6

Figure S2: The ^{13}C NMR spectrum of compound **2** in DMSO-d6

Figure S3: The DEPT-135 NMR spectrum of compound **2** in DMSO-d6

Figure S4: The HSQC spectrum of compound **2** in DMSO-d6

Figure S5: The COSY spectrum of compound **2** in DMSO-d6

Figure S6: The HMBC spectrum of compound **2** in DMSO-d6

Figure S7: HR-ESI-MS spectrum of compound **2** in the positive mode

Figure S8: HR-ESI-MS spectrum of compound **2** in the negative mode

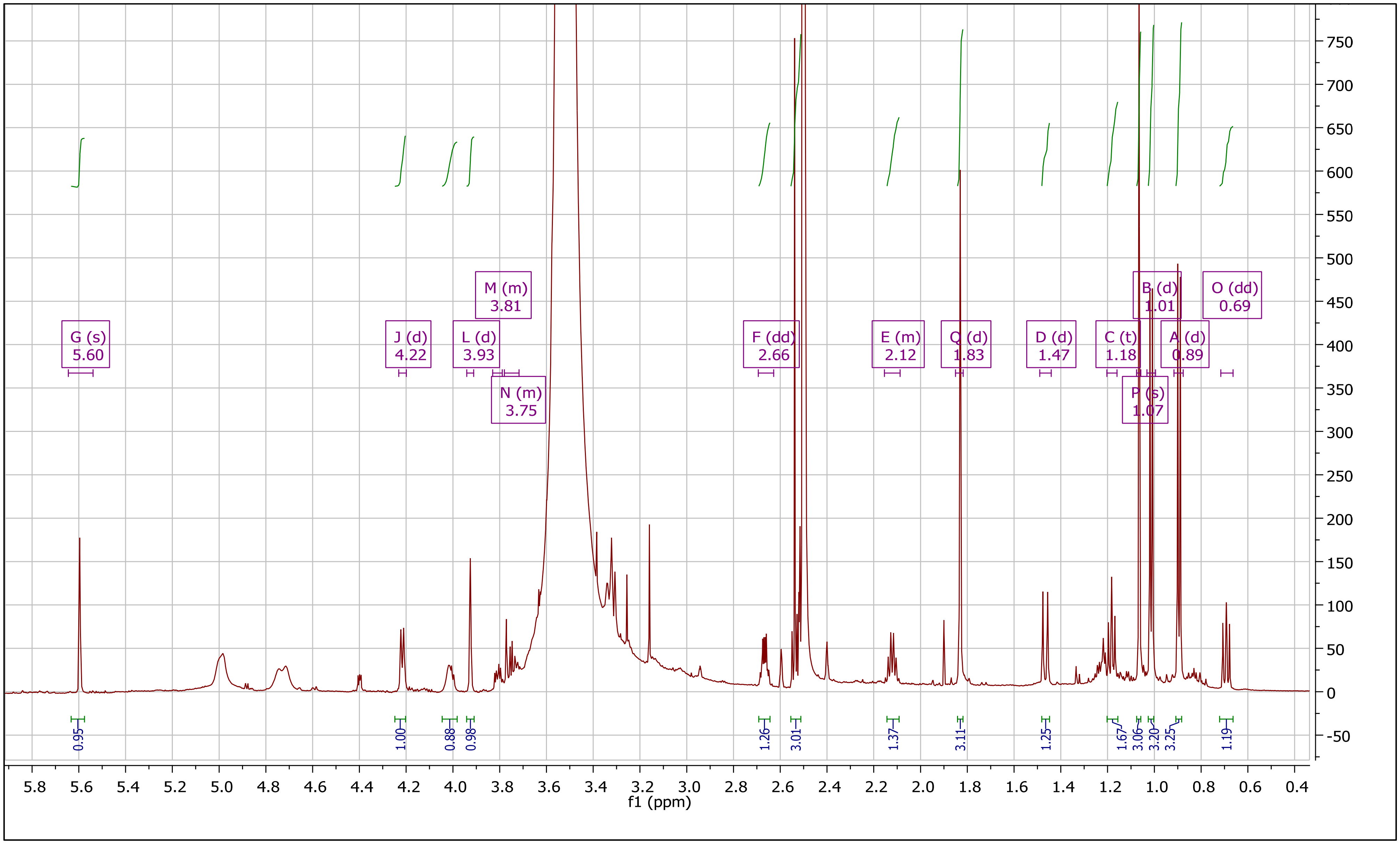

Figure S1: The ^{1}H NMR spectrum of compound **2** in DMSO-d6

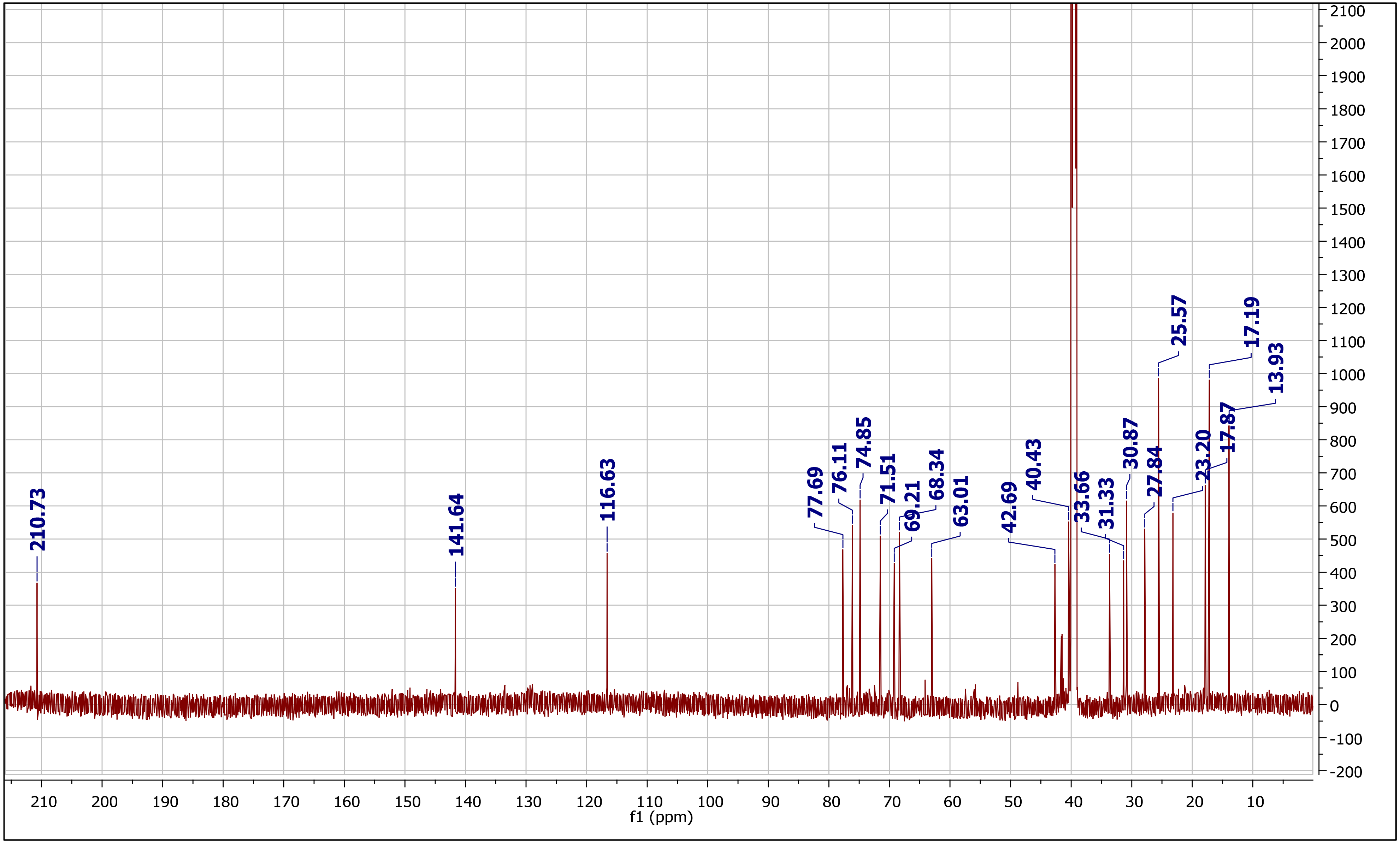

Figure S2: The ^{13}C NMR spectrum of compound **2** in DMSO-d6

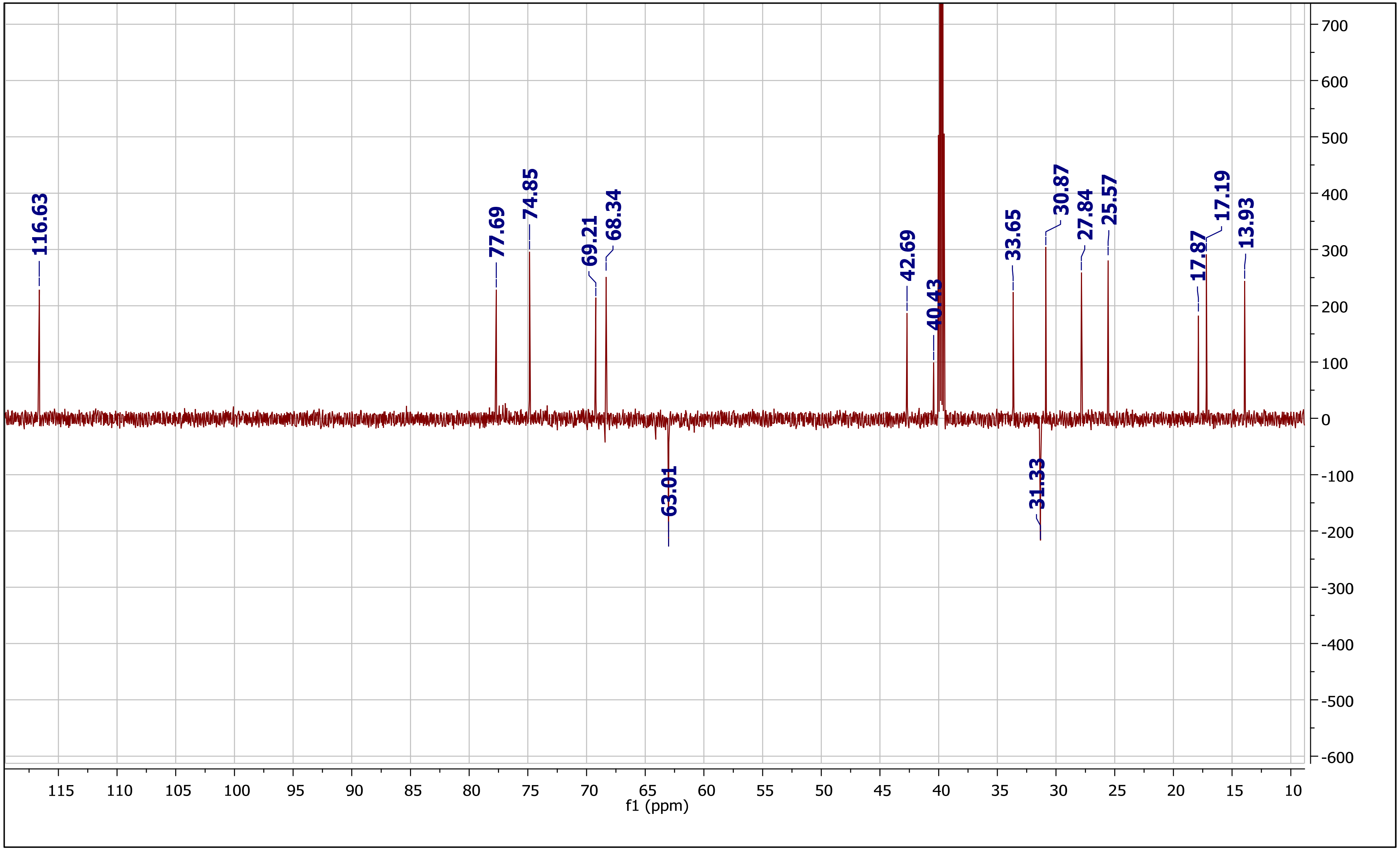

Figure S3: The DEPT-135 NMR spectrum of compound **2** in DMSO-d6

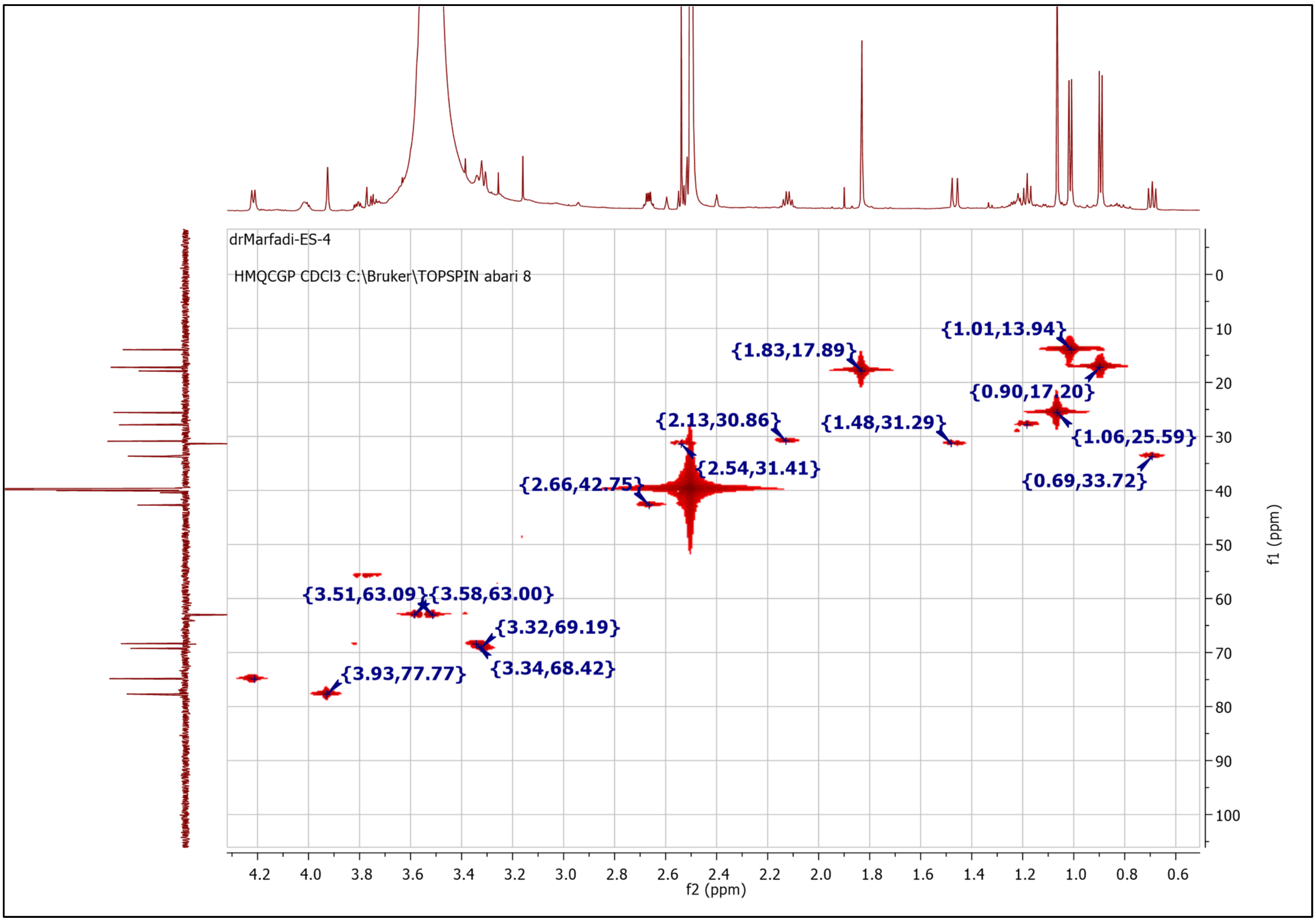

Figure S4: The HSQC spectrum of compound **2** in DMSO-d6

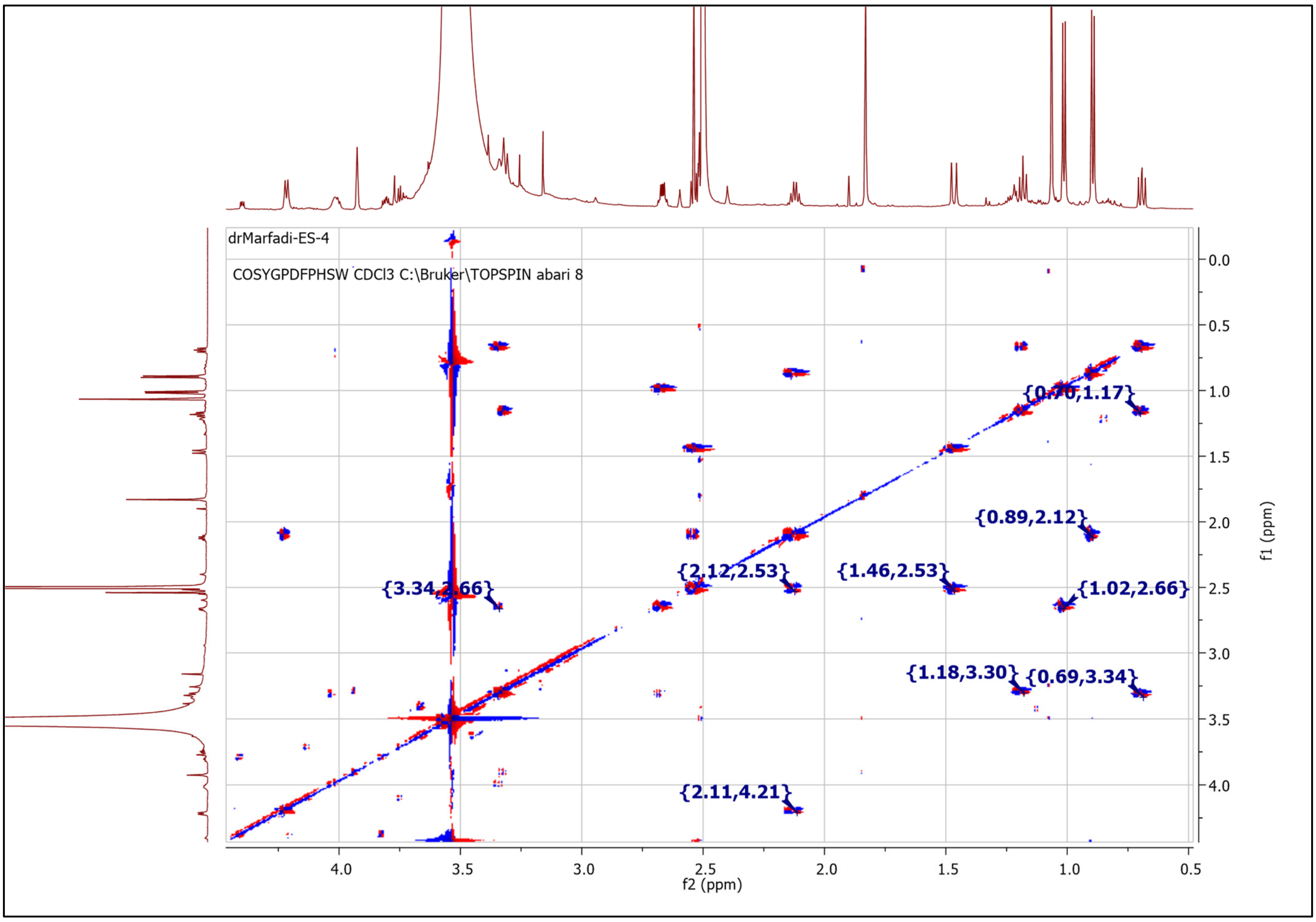

Figure S5: The COSY spectrum of compound **2** in DMSO-d6

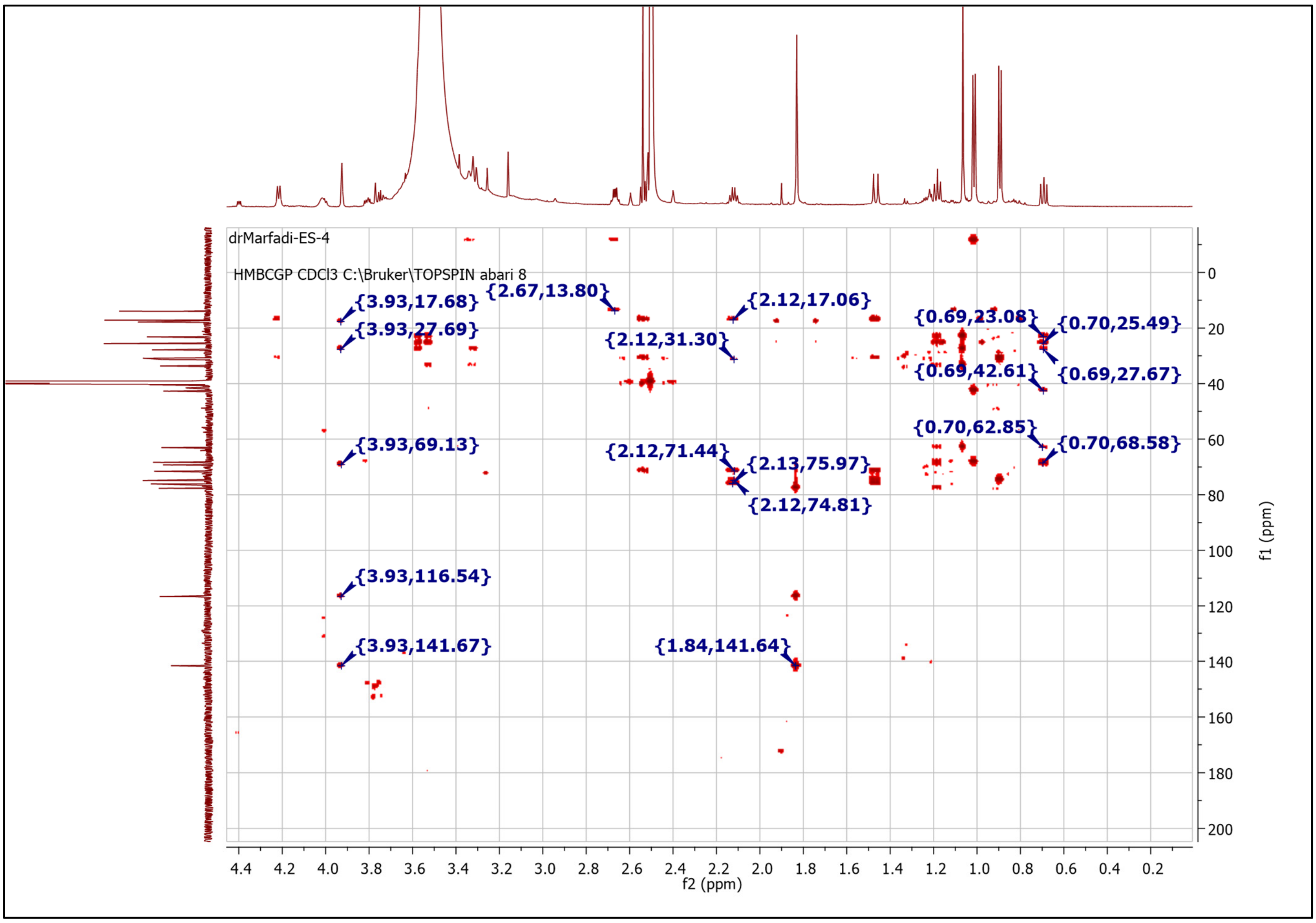

Figure S6: The HMBC spectrum of compound **2** in DMSO-d6

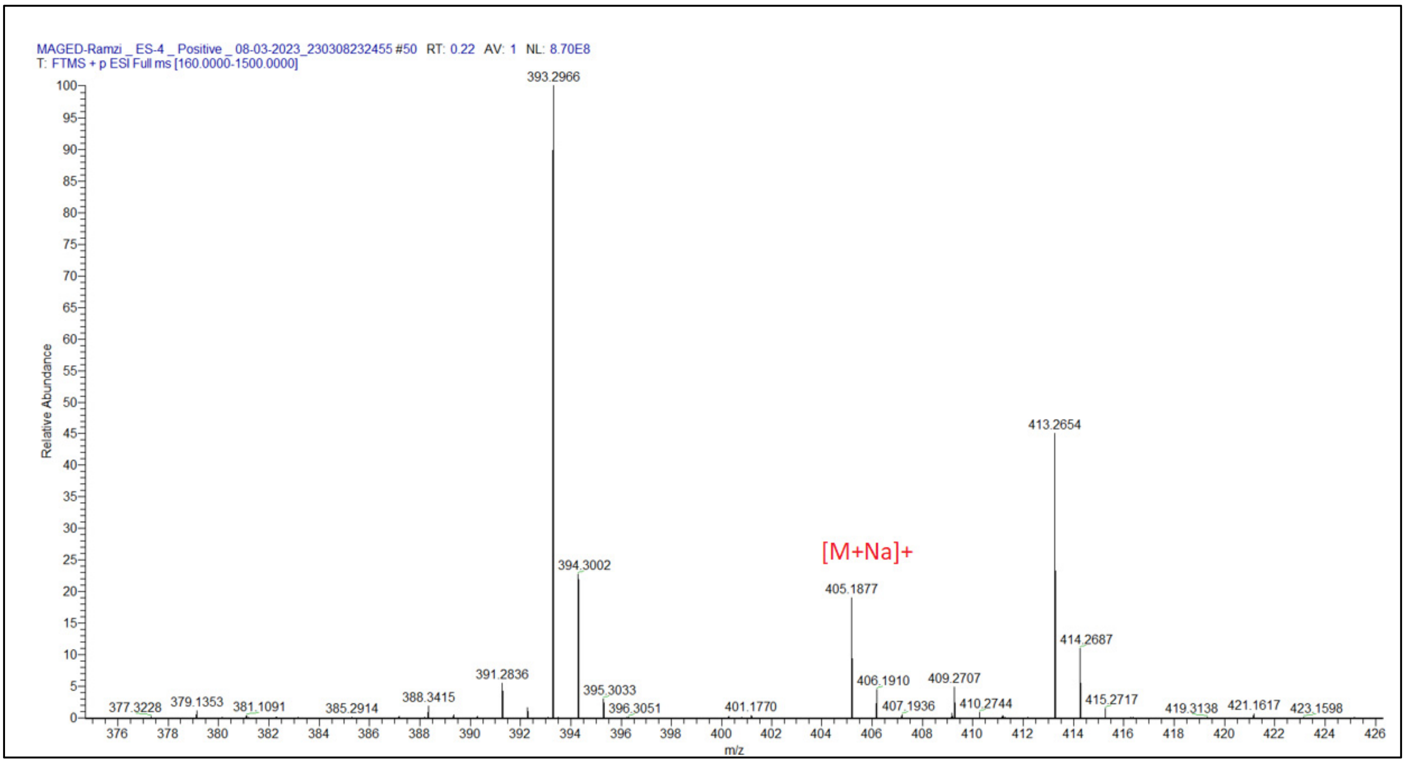

Figure S7: HR-ESI-MS spectrum of compound **2** in the positive mode

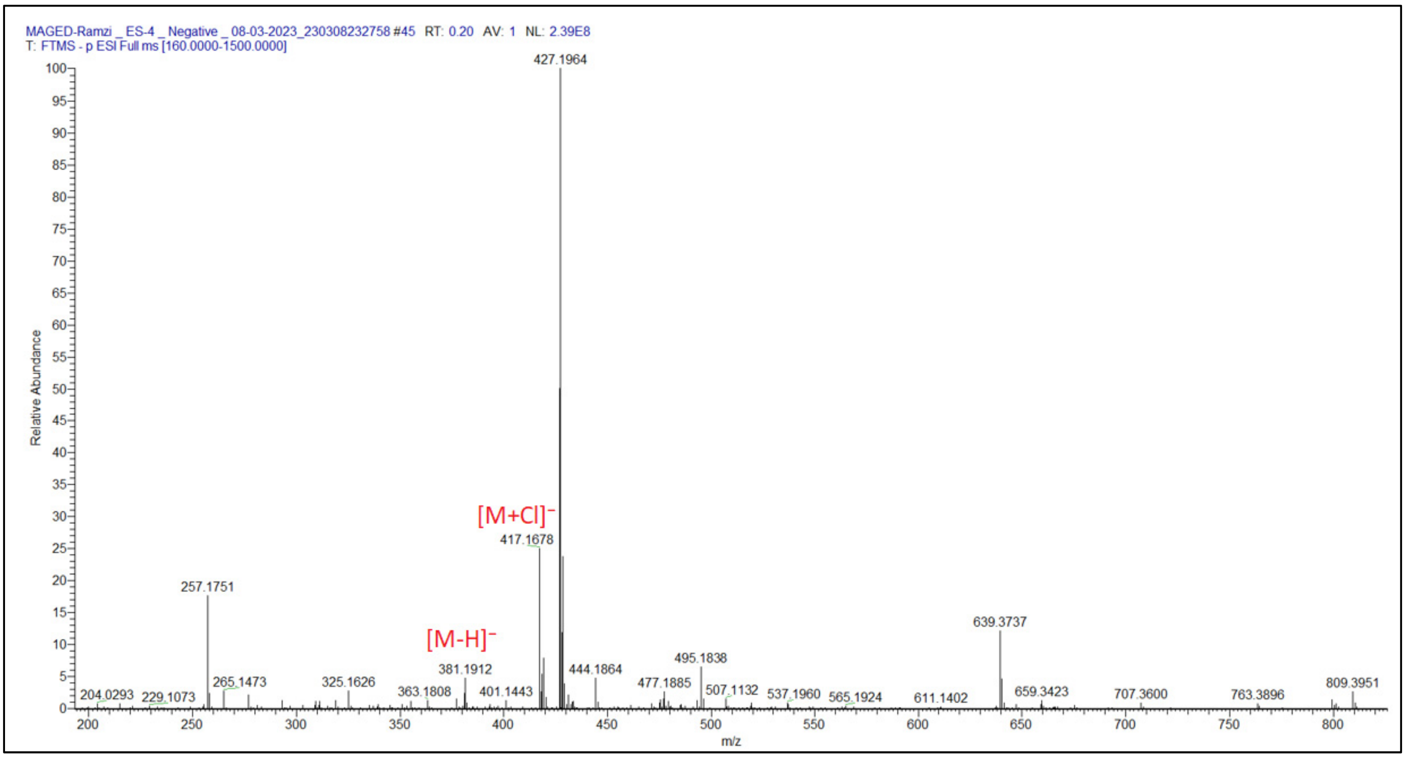

Figure S8: HR-ESI-MS spectrum of compound **2** in the negative mode
